# Development of an Orthotopic Patient-Derived Xenograft Model of Gastro-Esophageal Adenocarcinoma: A Single-Lineage Pilot Study

**DOI:** 10.3390/ijms27209051

**Published:** 2026-10-13

**Authors:** Rossella Reddavid, Maurizio Degiuli, Claudia Orrù, Silvia Giordano, Simona Corso

**Affiliations:** 1 Department of Oncology, University of Torino, 10124 Torino, Italy; 2 INOC—Istituto Nazionale Oncologico Candiolo, IRCCS, 10060 Candiolo (TO), Italy

**Keywords:** gastroesophageal junction adenocarcinoma, GEJ, gastric cancer, patient-derived xenograft, PDX, PDOX, orthotopic transplantation, metastasis, IVIS, precision oncology

## Abstract

Patient-derived xenografts (PDXs) preserve relevant histopathological and molecular characteristics of human tumors but, when implanted subcutaneously, poorly reproduce the native tumor microenvironment and patterns of metastatic dissemination. We developed a patient-derived orthotopic xenograft (PDOX) model of gastroesophageal junction (GEJ) adenocarcinoma based on the submucosal implantation of traceable tumor fragments. GFP/luciferase-labeled patient-derived GEJ adenocarcinoma organoids were propagated in mice as subcutaneous PDXs. Tumor fragments were then implanted into the gastric submucosal layer of seven NOD-SCID mice using a specifically developed surgical technique. Primary orthotopic tumor growth was qualitatively monitored longitudinally by in vivo IVIS imaging, whereas distant organ-associated bioluminescent signals were assessed ex vivo in excised organs at the experimental endpoint. Histopathological examination confirmed local orthotopic tumor growth. All procedures were completed without intraoperative or postoperative complications. Local tumor growth was observed in all xenografts. Distant organ-associated bioluminescent signals were detected in the liver and lungs, with an additional bowel-associated signal observed in one animal. These preliminary proof-of-concept findings demonstrate the technical feasibility of submucosal orthotopic implantation and longitudinal in vivo monitoring. However, given the small exploratory cohort and the use of a single patient-derived tumor lineage, the biological fidelity and generalizability of the model remain to be established in larger studies incorporating multiple independent tumors and comparative histopathological and molecular analyses.

## 1. Introduction

Gastric cancer is a biologically heterogeneous malignancy and remains a major cause of cancer-related morbidity and mortality worldwide [1,2]. Although surgery remains the cornerstone of curative treatment for localized disease, outcomes for locally advanced and metastatic gastric cancer continue to be limited by recurrence, metastatic progression, and therapeutic resistance [3,4,5]. The increasing integration of perioperative systemic therapy, molecularly targeted treatment, and immunotherapy has improved the therapeutic landscape, but clinically meaningful benefit remains strongly dependent on tumor biology and appropriate patient selection [6,7].

Molecular profiling has substantially changed the biological interpretation of gastric cancer. The Cancer Genome Atlas identified four major molecular subgroups—Epstein–Barr virus-positive (EBV), microsatellite instability (MSI), chromosomal instability (CIN), and genomically stable (GS) tumors—providing a framework for biomarker-driven investigation [8]. Consistent with this molecular complexity, our previous work has highlighted the importance of oncogene amplification, copy number, and co-occurring genomic alterations when selecting patients for molecular therapies [9,10,11,12]. Recent studies from the same translational platform have further shown that HER2, EGFR/EGFR-ligand, KRAS, and BRCA2 alterations can identify distinct therapeutic vulnerabilities in gastric and gastroesophageal cancer models [9,10,13,14,15,16].

Reliable experimental models are therefore essential for translating molecular observations into therapeutic hypotheses. Patient-derived xenografts (PDXs), generated by direct transplantation of human tumor material into immunodeficient mice, preserve important histopathological and genomic characteristics of donor tumors and provide a clinically informative platform for biomarker discovery and drug testing [17,18]. Within our research program, a comprehensive multilevel gastric cancer platform comprising PDXs, primary cell cultures, and three-dimensional organoids was established and molecularly characterized [19]. The collection captured the major histological and molecular subtypes of human gastric cancer, maintained substantial concordance with the corresponding primary tumors, and enabled the study of factors associated with engraftment and treatment response [19].

This platform has been used to address several precision-oncology questions. In HER2-driven gastric cancer, intensified HER2 blockade produced deeper and more durable responses than trastuzumab alone in selected highly amplified models [9]; in EGFR-addicted gastroesophageal adenocarcinoma, combined EGFR antibody and tyrosine-kinase inhibition increased therapeutic efficacy while the high expression of the EGFR ligands AREG and EREG has emerged as a potential biomarker of EGFR inhibitor sensitivity in gastroesophageal cancer lacking *EGFR* amplification [10,20]; *KRAS*-mutant gastric cancer models have demonstrated mutation-specific biological and pharmacological properties [21]; *BRCA2*-mutated tumors have shown responses to PARP inhibitors [13]. Together, these studies emphasize the value of patient-derived models for refining molecular selection rather than applying targeted agents to unselected populations.

Conventional subcutaneous PDXs nevertheless have an important biological limitation. Although they facilitate serial tumor measurement and controlled pharmacological experiments, tumors grow in an ectopic anatomical compartment and frequently fail to reproduce local invasion and spontaneous metastatic dissemination. Our systematic review of gastric cancer orthotopic models identified this limitation as a major rationale for the development of patient-derived orthotopic xenografts (PDOXs) [22]. Orthotopic implantation places tumor tissue within the organ of origin and may reproduce organ-specific tumor–stroma interactions, invasive growth, and metastatic behavior more faithfully than subcutaneous transplantation [22,23,24,25,26].

Published gastric cancer orthotopic models remain heterogeneous. In our previously published systematic review, only ten studies fulfilled predefined validation criteria; most models were derived from established cancer cell lines, approximately 90% used a subserosal implantation site, reported engraftment ranged from 0% to 100%, and complete biological validation was frequently lacking [22]. Other investigators have described glue-paste, suturing, and microsurgical orthotopic approaches, underscoring both the potential of the technique and the need for reproducible, standardized procedures [26,27,28,29,30,31]. These limitations led us to focus on two complementary methodological objectives: placement of viable tumor tissue within the gastric wall and reliable longitudinal tumor tracing in vivo.

We therefore developed a submucosal PDOX strategy using GFP/luciferase-labeled tumor material derived from our established patient-derived gastric cancer platform. This approach was specifically designed to combine a biologically relevant implantation site with non-invasive longitudinal imaging, while retaining the possibility of direct pathological confirmation. Importantly, the present series represents a preliminary proof-of-concept experiment rather than definitive validation of model performance.

The aim of this single-lineage pilot study was to assess the technical feasibility of submucosal orthotopic implantation, the ability of IVIS imaging to monitor local orthotopic tumor growth longitudinally in vivo, and the feasibility of detecting distant organ-associated bioluminescent signals by endpoint ex vivo imaging. Comprehensive biological validation, including comparative histopathological and molecular characterization across the different stages of model generation, as well as therapeutic and precision-oncology applications, was beyond the scope of the present study.

## 2. Results

### 2.1. Surgical Feasibility

Seven NOD-SCID mice underwent the definitive orthotopic surgical procedure with implantation of labeled tumor fragments derived from the precursor subcutaneous xenograft into the gastric submucosal layer. All seven procedures were completed successfully. No intraoperative or postoperative complications were reported in this preliminary cohort. These observations support the technical feasibility of the procedure; however, because the series included only seven animals, they should not be interpreted as definitive evidence of procedural safety or reproducibility but need to be performed in wider number of animals.

The observed proportion of successfully completed procedures was 100% (7/7; exact 95% CI, 59.0–100%), and the observed intraoperative and postoperative complication rates were both 0% (0/7; exact 95% CI, 0–41.0%). These wide confidence intervals reflect the substantial uncertainty associated with the small pilot cohort (Table 1 and Table S1, Figure 1).

### 2.2. Local Tumor Engraftment and Longitudinal In Vivo Imaging

Local orthotopic tumor growth was observed in all seven implanted animals. Although this corresponds to 100%, the value represents an exploratory observation from a very small cohort and should not be interpreted as the expected engraftment rate of the model in a broader population of tumors (Figure 2).

Serial in vivo IVIS imaging qualitatively detected the primary orthotopic tumor during follow-up. The representative imaging series shown in Figure 2 illustrates repeated detection of the luciferase-labeled primary tumor in an intact animal. Distant organ-associated signals were not evaluated as longitudinal in vivo endpoints and were assessed separately by ex vivo imaging of excised organs at the experimental endpoint.

### 2.3. Pathological Confirmation

After completion of longitudinal monitoring, pathological analysis was performed following animal sacrifice. Histopathological examination confirmed the tumor growth observed using IVIS (Figure 3), supporting concordance between in vivo imaging and post-mortem assessment. No site-specific histopathological confirmation of the distant organ-associated signals was performed.

### 2.4. Endpoint Ex Vivo Organ-Associated Bioluminescent Signals

At the experimental endpoint, ex vivo IVIS imaging of excised organs identified liver- and lung-associated bioluminescent signals in all seven animals, with an additional bowel-associated signal in one animal. These distant signals were not localized during the serial in vivo imaging of intact animals and were not confirmed by site-specific histopathology or a human-specific assay. They were therefore interpreted as organ-associated bioluminescent signals compatible with, but not definitive evidence of, tumor dissemination (Figure 4).

## 3. Discussion

The present study describes the development of a novel submucosal PDOX model of gastroesophageal junction adenocarcinoma and provides preliminary evidence supporting its technical feasibility and biological relevance. The study extends a translational research program in which patient-derived gastric cancer models have already been used for molecular classification and therapeutic investigation.

The model was conceived to address a major limitation of conventional subcutaneous patient-derived xenografts. Although PDXs preserve important histological and molecular features of the donor tumors [17,18,19], ectopic subcutaneous growth does not fully recapitulate the native tumor microenvironment and rarely reproduces spontaneous metastatic dissemination [22,25].

Importantly, the present study was not designed to directly compare subcutaneous and orthotopic implantation. No matched subcutaneous control cohort was included; therefore, no conclusion regarding superiority of the orthotopic approach in terms of engraftment, dissemination, or other biological outcomes can be drawn from the present data. The limited ability of subcutaneous PDXs to reproduce spontaneous metastatic dissemination is discussed solely as a literature-based rationale for the development of orthotopic models. A prospective matched comparison using the same patient-derived tumor lineage would be required to formally address this question.

Orthotopic transplantation provides a rational alternative as tumor material is implanted within the organ of origin. Experimental studies across multiple tumor types, including gastric cancer, have demonstrated that the anatomical site of implantation can profoundly influence tumor growth, invasion, and metastatic behavior [22,23,32,33,34].

Our previous systematic review highlighted substantial methodological heterogeneity in gastric PDOX models [22]. Most published studies relied on established tumor cell lines rather than patient-derived material; subserosal implantation predominated; engraftment rates varied widely, and several studies did not comprehensively address key criteria for model validation [22]. The present approach was developed specifically to address these limitations.

The model differs from previously described approaches in several important respects. First, tumor material was derived from a patient-derived gastric cancer experimental platform rather than from a conventional long-established cell line. This platform includes PDXs, primary cultures, and organoids and has demonstrated preservation of key histological and molecular characteristics of the corresponding donor tumors [19]. It has also supported molecularly selected therapeutic studies targeting HER2, EGFR, KRAS, BRCA2 and downstream signaling pathways.

Second, the model incorporated stable tumor labelling using luciferase. This feature is particularly valuable in orthotopic models because external caliper measurements, routinely used to monitor subcutaneous tumors, cannot be applied to lesions growing within the stomach. Bioluminescence imaging therefore enables longitudinal, non-invasive monitoring of tumor growth the same animal.

Third, we developed a submucosal implantation strategy rather than relying exclusively on superficial fixation of tumor tissue to the serosal surface. This approach was intended to more closely reproduce the anatomical relationship between gastro-esophageal tumors and the gastric wall while ensuring stable positioning of the xenograft.

The technical procedure proved feasible in the initial cohort. All seven animals underwent transplantation without reported intraoperative or postoperative complications. This finding is encouraging particularly because orthotopic gastric surgery in small animals can be technically challenging and prolonged procedures may increase perioperative morbidity.

The preliminary biological findings were also notable. Local tumor growth was observed in all seven animals. In addition, distant organ-associated bioluminescent signals were detected in association with the liver and lungs in all animals and with the bowel in one animal. However, since site-specific histopathological or human-specific validation was not performed, the available data cannot unequivocally establish that these signals represented metastatic lesions. In accordance with PDX-MI recommendations [35], future studies should include animal-by-animal histopathological assessment and the confirmation of human tumor origin using validated human-specific markers or molecular assays. Second, as the PDOX were generated from a single tumor lineage, this study demonstrates reproducibility of the surgical procedure within a specific tumor model rather than across the biological heterogeneity of gastro-esophageal adenocarcinoma. This distinction is particularly relevant for patient-derived models as gastro-esophageal adenocarcinomas display marked differences in histology, molecular drivers, stromal composition, and metastatic potential. A model intended for broad translational use should therefore be validated using multiple independent patient-derived tumors. Future studies should include intestinal, diffuse, and mixed histological subtypes and, where feasible, molecularly distinct tumors representing major genomic classes. Such an approach would allow the evaluation of whether PDOX engraftment and metastatic behavior are associated with specific biological characteristics of the donor tumor.

Third, since PDOXs have been generated in immunodeficient mice—immunodeficiency is essential for stable engraftment of human tumor tissue—it precludes the faithful recapitulation of a functional human immune microenvironment. This limitation is increasingly relevant given the central role of immunotherapy in the treatment of selected gastro-esophageal cancers, particularly tumors with microsatellite instability or other immunogenic features.

Fourth, although orthotopic implantation improves anatomical fidelity, human stromal components are progressively being replaced by murine elements during xenograft expansion and serial passage.

An additional limitation of the present study is the absence of a direct and synchronous comparison between subcutaneous and orthotopic implantation using the same luciferase-labeled tumor model. Future validation should therefore include larger cohorts and prospective matched comparisons of subcutaneous and orthotopic implantation of the same patient-derived tumor model, with comprehensive assessment of histopathological and molecular fidelity, metastatic behavior, and therapeutic response.

Finally, sex-related differences were not assessed, potentially limiting the generalizability of our findings.

An important strength of the present study is the use of longitudinal imaging. IVIS enabled repeated, non-invasive qualitative assessment of the primary orthotopic tumor in vivo, whereas distant organ-associated signals were identified only by endpoint ex vivo imaging of excised organs. This approach could provide quantitative longitudinal endpoints and facilitate the evaluation of treatment response and metastatic progression in future therapeutic studies.

The present findings should therefore be regarded as hypothesis-generating rather than definitive. They demonstrate that submucosal implantation of traceable patient-derived tumor material is technically feasible and capable of producing reproducible local orthotopic tumor growth and endpoint organ-associated bioluminescent signals in this preliminary series. However, they do not provide definitive validation of the model.

A larger prospective validation study should include multiple independent tumor lines and should prospectively define operative mortality, perioperative complications, engraftment rate, time to tumor detection, metastatic incidence, anatomical distribution of metastases, and concordance between imaging and pathology. Direct comparison with matched subcutaneous PDXs would also be particularly informative to determine whether orthotopic implantation enhances metastatic dissemination or alters the pattern of metastatic spread.

Overall, the present proof-of-concept study provides a methodological foundation for development of a larger gastro-esophageal PDOX platform. If validated in expanded cohorts, this model could complement existing patient-derived experimental systems and facilitate studies of disease progression, metastasis, therapeutic resistance, and precision oncology.

## 4. Materials and Methods

### 4.1. Study Design

The experimental study was designed as a single-lineage pilot study to assess the technical feasibility of a traceable orthotopic xenograft model of gastroesophageal junction adenocarcinoma derived from an existing molecularly annotated patient-derived platform [15]. The primary objectives were to assess surgical feasibility, local orthotopic tumor engraftment, and longitudinal monitoring by IVIS. The seven orthotopic recipient mice represented host-level experimental units derived from a single patient-derived tumor lineage; therefore, model-level biological replication was *n* = 1. No matched subcutaneous control group was included because the study was designed to assess technical feasibility rather than to compare the performance of orthotopic and subcutaneous implantation. Comparisons with subcutaneous PDXs are therefore based exclusively on previously published evidence (Figure 5).

No formal a priori sample-size calculation was performed because this was an exploratory technical-feasibility pilot study. Seven mice were included in the orthotopic cohort. All animals undergoing orthotopic implantation were included in the analysis, with no exclusions or attrition.

No additional prespecified inclusion or exclusion criteria were applied after orthotopic implantation.

### 4.2. Patient-Derived Tumor Model and Provenance

The GTR0233 model was derived from a gastroesophageal junction (GEJ) adenocarcinoma obtained from a male Caucasian patient. The primary tumor was a grade 2, pathological stage II tumor collected at surgery without previous neoadjuvant treatment. Molecular characterization identified *HER2* copy-number gain and classified the tumor as chromosomal instability (CIN), microsatellite stable (MSS), and Epstein–Barr virus (EBV)-negative. GTR0233 belongs to the previously established and molecularly characterized gastric cancer PDX collection [19].

Organoids were derived from the second in vivo PDX passage (P2). Detailed culture/passaging intervals and specific selection or enrichment procedures for reporter-positive cells were not recorded in the available experimental documentation. STR profiling was performed on DNA extracted from the in vitro-derived cells using Cell ID (Promega, Madison, WI, USA). Sixteen STR markers were evaluated and showed concordance with the original tumor lineage. STR authentication was not repeated at each subsequent propagation stage. The longitudinal stability of GFP/luciferase reporter expression and post-orthotopic phenotypic fidelity were not assessed in the present study. The present study therefore represents a single independent patient-derived tumor lineage.

### 4.3. Experimental Animals

Male NOD-SCID mice aged at least 9 weeks old were obtained from Charles River Laboratories and used as recipient animals. Male animals were preferentially selected because their larger body size and robustness were considered advantageous for the surgical procedure. Animals were maintained under institutional animal-care conditions. All animal procedures were approved by the Ethical Commission of INOC and the Italian Ministry of Health (n° 58/2021-PR). Animal housing, husbandry, and acclimatization were conducted according to the institutional animal-care procedures approved within the authorized experimental protocol. Individual baseline body weights were not available for retrospective quantitative reporting.

### 4.4. Preoperative Preparation

Enrofloxacin (Baytril 2.5%, 25 mg/mL) was administered subcutaneously. According to the operative protocol, 40 µL of stock solution was diluted to a final volume of 2 mL with sterile physiological saline, and 200 µL of the resulting preparation was administered per mouse, corresponding to 0.1 mg of enrofloxacin per mouse. The protocol indicated a target dose of 5 mg/kg; however, individual baseline body weights were not available for retrospective verification of the exact weight-adjusted dose received by each animal. The first prophylactic dose was administered on the day before surgery, followed by approximately 24-hourly administration starting on the day of surgery and continuing for 5 days postoperatively. Postoperative analgesia with meloxicam (5 mg/kg) was administered for 5 days.

The abdominal surgical area was shaved. On the day of surgery, animals were fasted for 4 h, with water available ad libitum, to ensure an empty stomach and facilitate gastric manipulation, implantation, and suturing. Surgical instruments were sterilized before use, and orthotopic implantation was performed under aseptic conditions in a laminar-flow hood. Mice were anesthetized with 2.5% isoflurane in oxygen and maintained on a heated surface during surgery to prevent hypothermia. Anesthetic depth was assessed by monitoring the respiratory pattern and absence of the pedal withdrawal reflex. The duration of anesthesia was minimized as much as possible.

### 4.5. Generation of GFP/Luciferase-Expressing Tumor Cells

GTR0233 organoids, derived from the P2 PDX, were transiently dissociated using TrypLE 1×. Cells were seeded at a density of 1 × 10^6^ cells per 100-mm culture dish and stably transduced with lentiviral particles at a concentration corresponding to 40 ng/mL p24 using the pRRLsIn.PPT.CMV.Luciferase.IresEMCwt.eGFP.pre construct. Successful transduction and functional luciferase expression were assessed by bioluminescence, quantified using a VICTOR X Multilabel Plate Reader (PerkinElmer, Waltham, MA, USA). The genetic identity of the in vitro-derived material relative to the original tumor was confirmed by STR profiling (Cell ID, Promega).

### 4.6. Establishment of Traceable Subcutaneous Xenografts

Before in vivo implantation, organoids were dissociated using TrypLE 1× in order to minimize damage. Dissociated organoid-derived cells were injected subcutaneously into immunodeficient mice at approximately 5 × 10^5^ cells per mouse. Tumor establishment was evaluated approximately one month later by using caliper measurements and IVIS (Figure 1).

A single mouse was used to generate the precursor subcutaneous xenograft from which the tumor fragments for the seven orthotopic recipients were obtained. Consequently, the seven PDOX-bearing recipient mice represent host-level experimental units derived from one independent patient-derived tumor lineage rather than independent biological tumor models.

### 4.7. Preparation of Tumor Fragments

The precursor subcutaneous xenograft was surgically excised and manually sectioned into tumor fragments with a target size of approximately 2 × 2 × 2 mm, within an acceptable range of approximately 1–2 mm per dimension, according to the surgical protocol. Fragment size was kept as uniform as technically feasible across the seven orthotopic recipient mice (Figure 1).

### 4.8. Surgical Procedure

Following induction of anesthesia, mice were positioned for abdominal surgery. A midline incision of the upper abdomen was performed through the skin, muscular fascia, and peritoneum. A surgical retractor was positioned, and the stomach was identified and gently exteriorized.

The posterior gastric wall near the greater curvature was selected as the implantation site for technical and animal-welfare considerations. Although the GTR0233 donor tumor originated from the GEJ, implantation closer to the cardia/GEJ was avoided because of the potential for early interference with gastric transit and food intake. The selected site provided greater anatomical compliance for implantation and was considered less likely to cause early obstruction or feeding impairment. Moreover, the posterior gastric wall was considered less exposed to gravitational and mechanical stress associated with gastric contents, taking into account the natural quadrupedal posture of the mouse. Thus, the present model represents gastric-wall implantation of GEJ-derived tumor material; anatomical equivalence between the implantation site and the donor GEJ site was not assessed. A purse-string suture was positioned around the intended implantation area.

A small incision was made, and approximately 10 µL of sterile physiological saline was gently injected into the gastric wall using a 1-mL graduated syringe to separate the muscular and submucosal layers. The resulting submucosal pocket was enlarged with microsurgical scissors. A viable tumor fragment was introduced into the pocket, avoiding perforation of the gastric mucosa. The purse-string suture was then tightened to completely close the pocket and retain the xenograft within the gastric wall. The stomach was returned to the abdominal cavity. The abdominal wall and skin were subsequently closed. 5-0 polypropylene was used for peritoneal closure and 5-0 silk for skin closure; Vicryl 4-0/5-0 on a round needle was used for surgical suturing (Figure 1).

### 4.9. Postoperative Care

Animals were placed on a heated surface during recovery. Animals were fasted for approximately 24 h after surgery, with water available ad libitum, to minimize mechanical stress on the operated stomach and gastric sutures during the initial postoperative period. Easily digestible moist food was subsequently placed on the cage floor to facilitate food reintroduction. Following surgery, animals were maintained in a heated environment during the initial recovery period. Antibiotic and analgesic treatment was continued for 5 days postoperatively, with meloxicam administered at 5 mg/kg. Animals were clinically monitored five times per week during subsequent follow-up.

For reporting purposes, intraoperative complications were defined as unexpected events occurring during anesthesia or surgery that required modification or interruption of the planned procedure. Postoperative complications were defined as clinically relevant adverse events occurring during the first postoperative week that required additional treatment, veterinary intervention, or premature termination of the experiment beyond the supportive care specified in the approved protocol.

### 4.10. Welfare Monitoring and Humane Endpoints

Animal welfare was prospectively assessed according to the approved experimental protocol using predefined clinical parameters, including skin and coat condition, mucosal appearance, muscle tone, posture, locomotor activity, respiration, behavior, feces and urine, and body weight. Each parameter was graded according to increasing severity. Specific humane endpoints included generalized piloerection, ulceration or dehydration, cyanosis or jaundice, absence of reflexes or muscle spasms, permanent lateral recumbency or inability to maintain quadrupedal posture, severe abdominal distension, inability to move when stimulated, severe dyspnea, complete loss of reflexes, blood in feces or urine, and body-weight loss > 20%.

### 4.11. Euthanasia

At the experimental endpoint or when predefined humane endpoints were reached, animals were euthanized according to the approved animal protocol. Deep anesthesia was induced with 3% isoflurane in oxygen, followed by cervical dislocation.

### 4.12. In Vivo Bioluminescence Imaging

Serial bioluminescence imaging was performed initially approximately every two weeks, and then approximately weekly during the final phase using an IVIS Spectrum CT system (Revvity, Waltham, MA, USA). D-luciferin (PerkinElmer; 15 mg/mL, 200 µL per mouse) was administered by subcutaneous injection. Following luciferin administration, animals were placed in the anesthesia induction chamber for 3 min and subsequently positioned prone in the imaging system. Images were acquired using Diffuse Luminescence Imaging Tomography, with automatic exposure (50–20 ms), a field of view (FOV) of 6.6, and binning of 8. Each acquisition lasted approximately 5 min. Animals were not depilated for bioluminescence imaging. No background subtraction was applied, and no saturated pixels were observed. IVIS imaging was used qualitatively to assess the serial detection of luciferase-labeled tumor tissue; quantitative longitudinal total-flux or radiance analyses were not performed.

### 4.13. Assessment of Distant Organ Bioluminescent Signals

Serial in vivo IVIS imaging was used for qualitative assessment of the primary orthotopic tumor. Distant organ-associated bioluminescent signals were assessed separately by ex vivo IVIS imaging of excised organs at the experimental endpoint. Ex vivo organ positivity was assessed qualitatively based on the presence of a detectable luciferase-associated signal. No background subtraction was performed. Because site-specific histopathological or human-specific confirmation was not performed, these organ-associated signals were not classified as histologically confirmed metastases.

### 4.14. Pathological Analysis

Pathological examination was performed after animal sacrifice. The pathological assessment confirmed the local tumor growth detected by IVIS.

### 4.15. Statistical Analysis

Given the exploratory nature of this single-lineage pilot study, outcomes were summarized descriptively. No formal a priori sample-size calculation was performed. Observed proportions are reported together with two-sided exact binomial 95% confidence intervals (Clopper–Pearson method) to illustrate the uncertainty associated with the small sample size. No hypothesis testing or comparative statistical analysis was performed. The seven orthotopic recipient mice were considered host-level experimental units, whereas model-level biological replication was n = 1.

## 5. Conclusions

We developed a novel submucosal patient-derived orthotopic xenograft model of gastroesophageal junction adenocarcinoma integrating traceable tumor cells, organoid culture, conventional PDX expansion, orthotopic gastric implantation, and longitudinal in vivo imaging.

In this preliminary cohort of seven NOD-SCID mice, orthotopic implantation was technically feasible and resulted in local tumor growth in all animals. Longitudinal in vivo IVIS imaging qualitatively demonstrated local orthotopic tumor growth, while endpoint ex vivo imaging identified distant organ-associated bioluminescent signals. However, because these distant signals were not confirmed by site-specific histopathological or human-specific analyses, no definitive conclusions regarding metastatic capability could be drawn. Larger prospective studies incorporating rigorous pathological and molecular validation of distant lesions are required before the biological and translational validity of the model can be established.

## Figures and Tables

**Figure 1:**
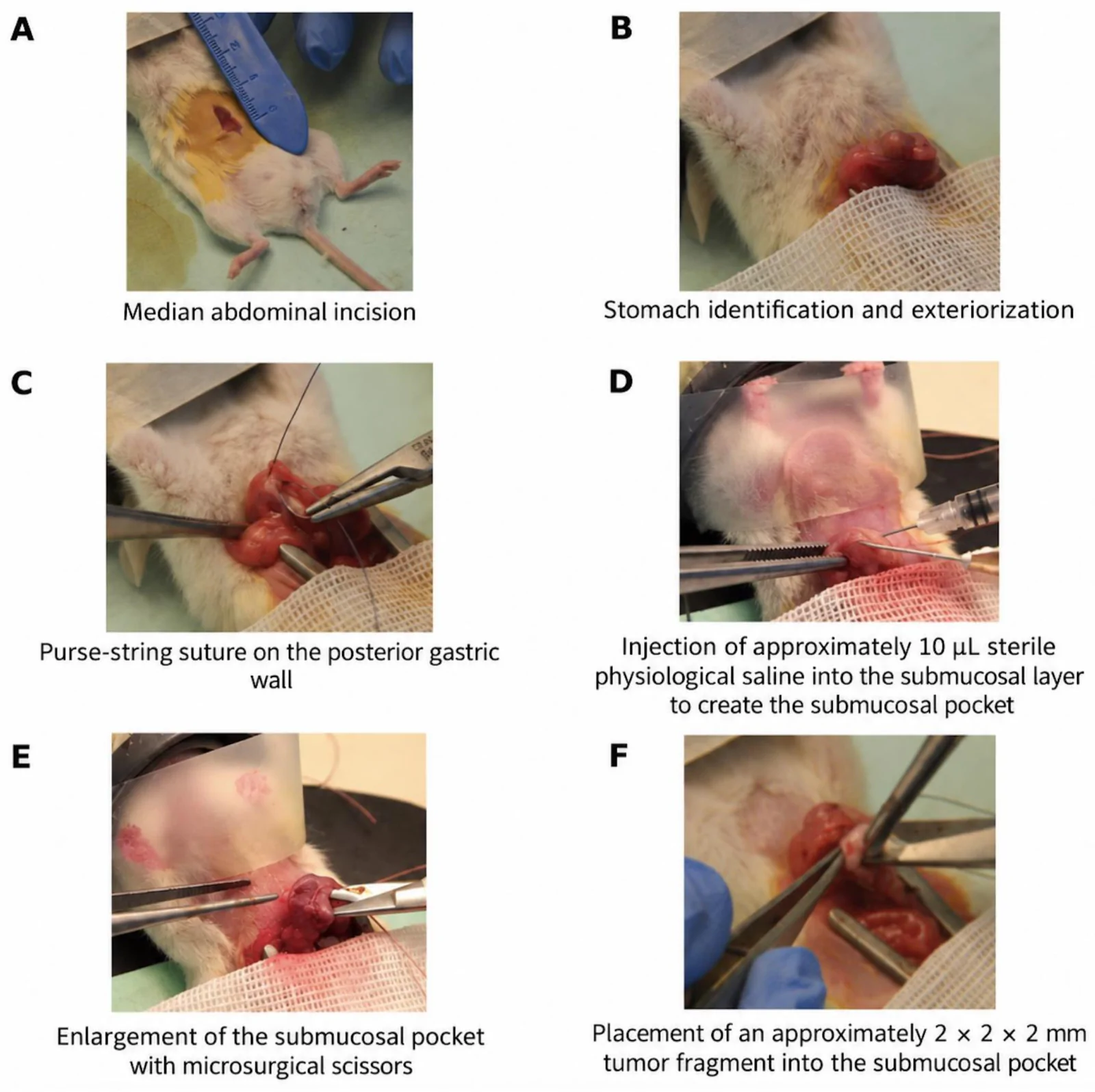
**Surgical procedure for submucosal orthotopic implantation of GTR0233 tumor fragments.** Following a midline upper abdominal incision, the stomach was gently exteriorized and a purse-string suture was placed on the posterior gastric wall near the greater curvature. Physiological saline was injected between the muscular and submucosal layers to facilitate creation of a submucosal pocket, which was enlarged using microsurgical scissors. A viable GTR0233 tumor fragment (approximately 2 × 2 × 2 mm), obtained from the precursor subcutaneous xenograft, was inserted into the submucosal pocket, and the purse-string suture was tightened to secure the fragment within the gastric wall. The stomach was subsequently returned to the abdominal cavity and the abdominal wall was closed. Images illustrate the surgical procedure used for the seven orthotopic recipient mice.

**Figure 2:**
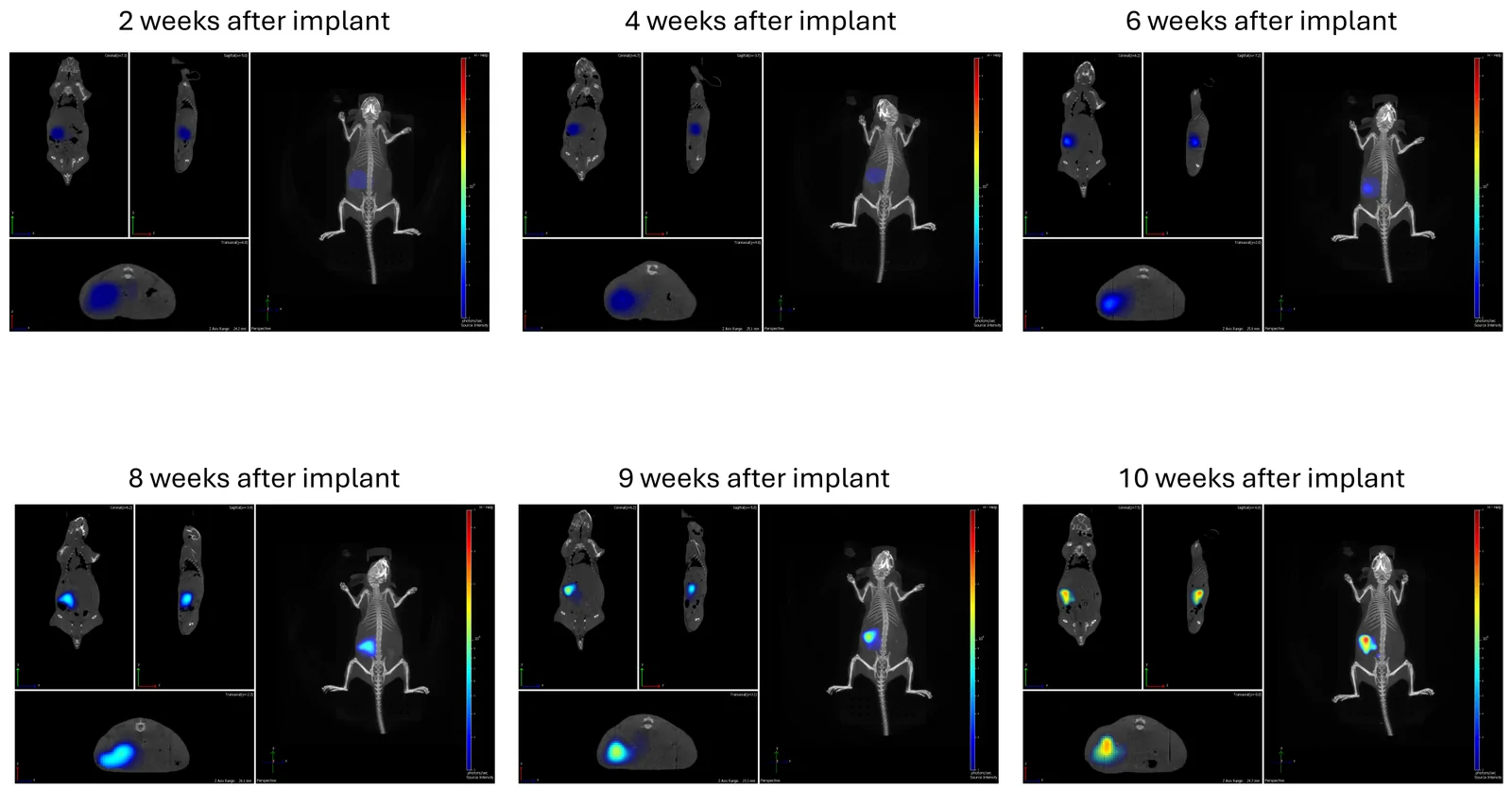
**Representative longitudinal in vivo IVIS imaging of the primary orthotopic tumor in PDOX-2.** Serial images obtained at the recorded time points of 2, 4, 6, 8, 9, and 10 weeks after implantation illustrate repeated qualitative detection of luciferase-labeled primary tumor tissue. Imaging was initially performed at approximately two-week intervals and was shortened to approximately weekly intervals during the final phase because of deterioration in the animal’s clinical condition. Exact post-implantation days could not be reliably recovered from the available historical records and were therefore not retrospectively inferred. Images are presented for qualitative visualization only and do not represent quantitative measurements of tumor growth.

**Figure 3:**
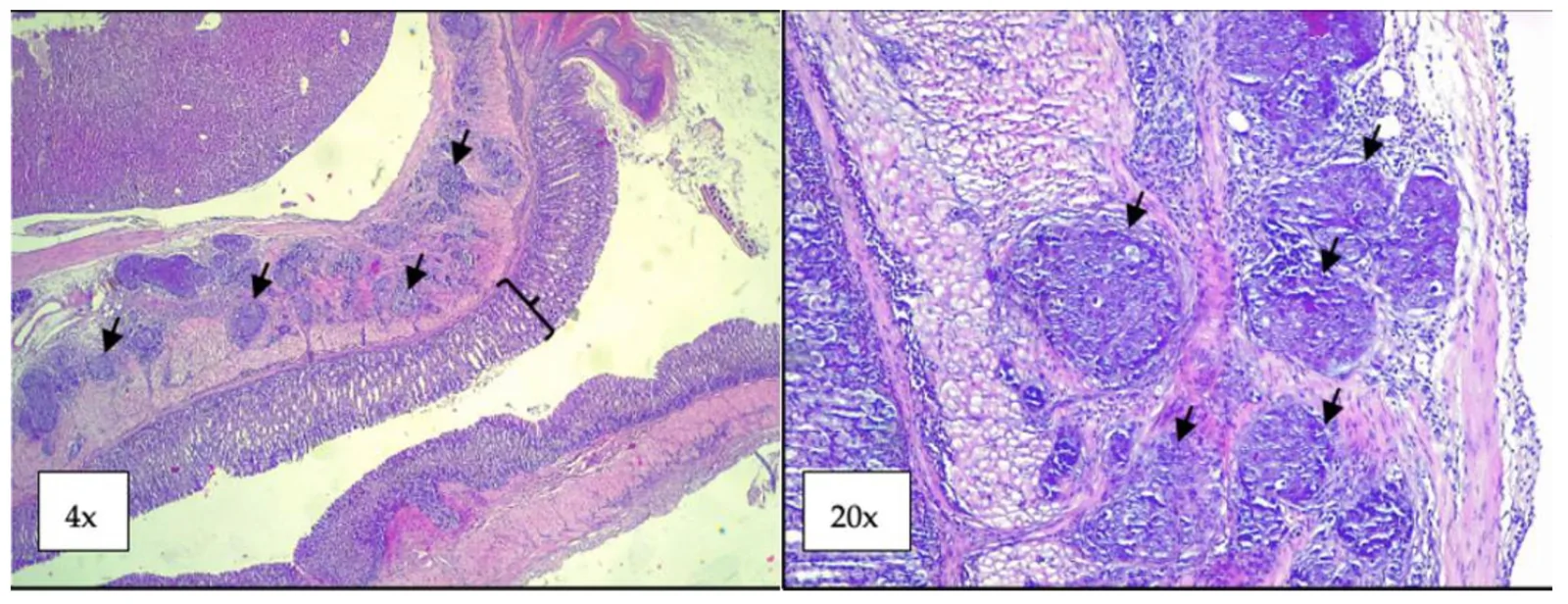
**Histopathological confirmation of local orthotopic tumor growth.** Representative hematoxylin and eosin (H&E)-stained sections of the primary gastric lesion, shown at 4× and 20× magnification. Arrowheads indicate tumor foci within the gastric submucosa, whereas the bracket delineates the adjacent normal gastric mucosa. Stomachs from all seven mice were examined histopathologically. These sections document local orthotopic tumor growth. The animal identifier of the representative specimen could not be reliably recovered from the available historical records. Calibrated length information could not be recovered from the original historical pathology files; therefore, scale bars were not retrospectively reconstructed.

**Figure 4:**
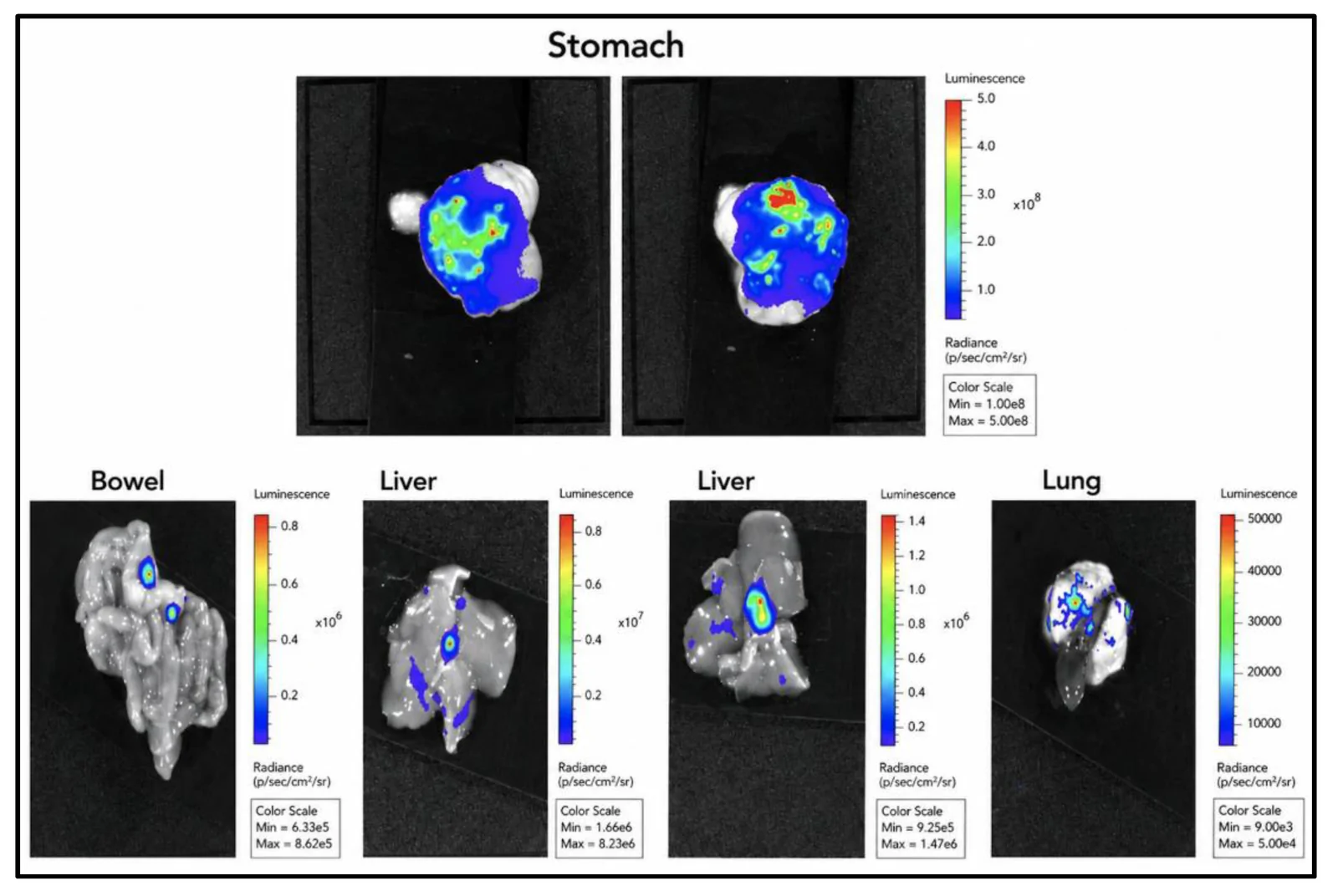
**Representative endpoint ex vivo organ-associated bioluminescent signals from PDOX-2.** IVIS imaging of excised organs showed liver-, lung-, and bowel-associated bioluminescent signals. The two liver panels represent acquisitions of the two sides of the same liver. Organ images were displayed using independently adjusted luminescence scales and therefore cannot be quantitatively compared across organs. These signals were not confirmed by site-specific histopathology or a human-specific assay.

**Figure 5:**
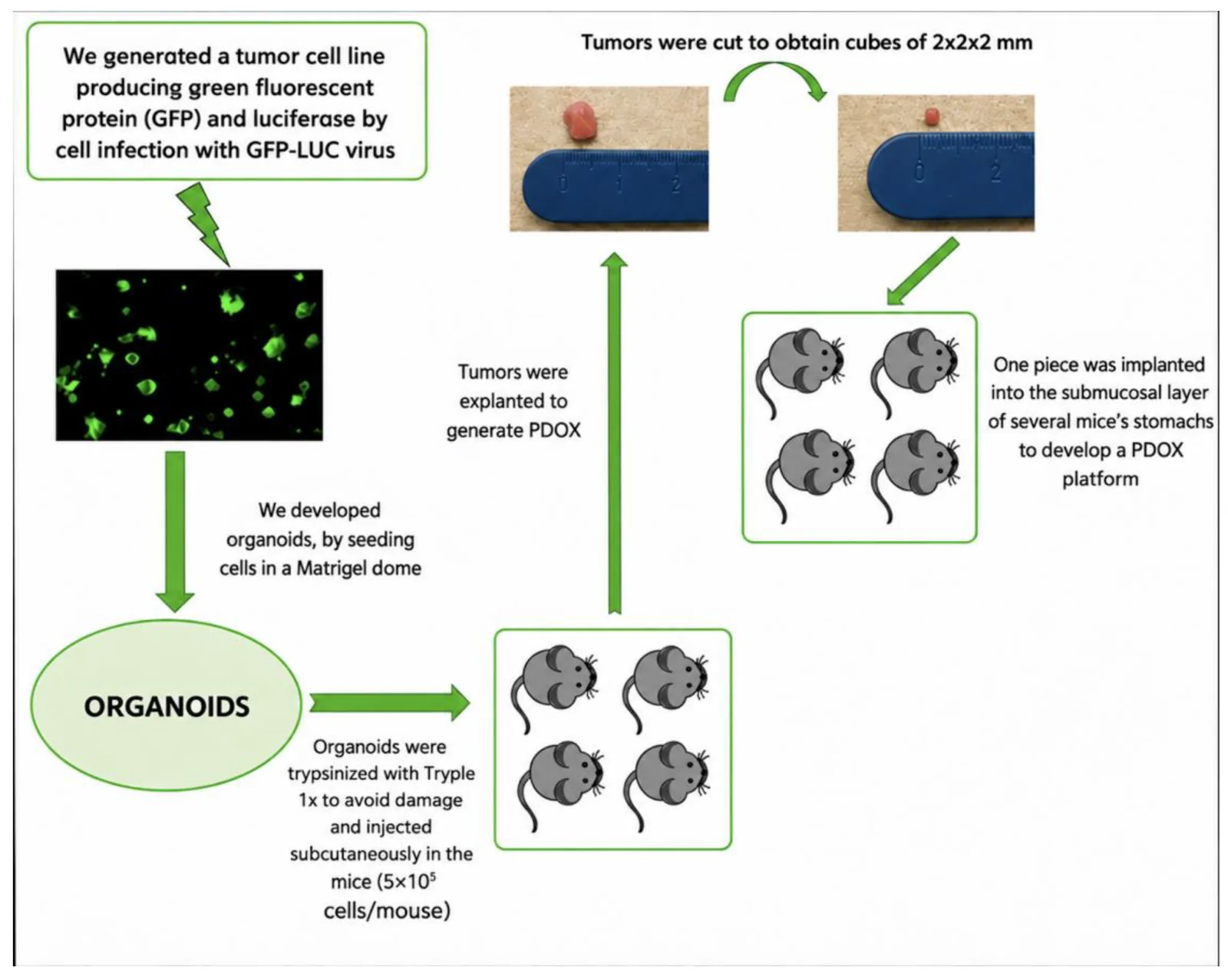
**Experimental workflow for generation of the GEJ adenocarcinoma PDOX model**. GFP/luciferase-expressing tumor cells were generated and expanded as three-dimensional organoids. Organoid-derived cells were implanted subcutaneously to establish traceable PDXs. Tumors were subsequently explanted, sectioned into small fragments, and implanted within the gastric submucosal compartment to generate PDOXs.

**Table 1:** Preliminary Experimental Outcomes of the PDOX Cohort.

Outcome	Observation	Assessment/Confirmation Method	Animals Assessed
Local orthotopic tumor growth	7/7 (100%; 95% CI, 59.0–100%)	In vivo IVIS	7
Local gastric tumor confirmation	7/7	H&E histopathology	7
Liver-associated signal	7/7 (100%; 95% CI, 59.0–100%)	Ex vivo IVIS	7
Liver histopathological confirmation	Not performed	No site-specific H&E/human-specific assay	0
Lung-associated signal	7/7 (100%; 95% CI, 59.0–100%)	Ex vivo IVIS	7
Lung histopathological confirmation	Not performed	No site-specific H&E/human-specific assay	0
Bowel-associated signal	1/7 (14.3%; 95% CI, 0.4–57.9%)	Ex vivo IVIS	7
Bowel histopathological confirmation	Not performed	No site-specific H&E/human-specific assay	0

**Note:** All proportions and exact two-sided 95% confidence intervals (Clopper–Pearson method) are descriptive host-level observations conditional on the single patient-derived tumor lineage and experimental preparation. They should not be interpreted as validation across independent patient-derived tumors or as definitive estimates of model performance. Distant organ-associated IVIS findings represent ex vivo bioluminescent signals and not histologically confirmed metastases.

## Data Availability

Data supporting this study are available from the corresponding author upon reasonable request, subject to institutional and ethical restrictions. Available materials include retained animal-level experimental timelines and outcomes, available IVIS acquisition metadata, original uncropped IVIS and ex vivo organ images, available histopathology images of the primary orthotopic tumors, model-provenance and model-passport information for GTR0233, and the surgical and animal-care protocols used in the study. Quantitative longitudinal total-flux/radiance datasets are not available because IVIS imaging was used qualitatively in this pilot study and these quantitative source data were not collected. Patient-related information will be provided only in de-identified form and in accordance with applicable ethical approvals, informed-consent provisions, and institutional data-protection requirements.

## Supplementary Materials

The following supporting information can be downloaded at: https://www.mdpi.com/article/10.3390/ijms27209051/s1.

## Abbreviations

The following abbreviations are used in this manuscript:

GC	Gastric cancer
GEJ	Gastroesophageal junction
PDX	Patient-derived xenograft
PDOX	Patient-derived orthotopic xenograft
GFP	Green fluorescent protein
GFP-LUC	Green fluorescent protein–luciferase
IVIS	In Vivo Imaging System
H&E	Hematoxylin and eosin
IHC	Immunohistochemistry
NOD-SCID	Non-obese diabetic/severe combined immunodeficiency
MSI	Microsatellite instability
MSS	Microsatellite stable
TCGA	The Cancer Genome Atlas
EGFR	Epidermal growth factor receptor
HER2	Human epidermal growth factor receptor 2
RTK	Receptor tyrosine kinase
KRAS	Kirsten rat sarcoma viral oncogene homolog
mTOR	Mechanistic target of rapamycin
