# Supplementary material for: Development of an Orthotopic Patient-Derived Xenograft Model of Gastro-Esophageal Adenocarcinoma: A Single-Lineage Pilot Study
Source: Int J Mol Sci. 2026 Oct 13;27(20):9051. doi: 10.3390/ijms27209051 (PMC13704638; doi:10.3390/ijms27209051)
Supplement: Supplementary file 1 [file ijms-27-09051-s001.zip › ijms-4547331-supplementary.pdf]

International Journal of
Molecular Sciences

MDPI

Table S1. Individual Animal-Level Outcomes in the PDOX Pilot Cohort.

Animal	Follow-up	Endpoint/reason	Local in vivo IVIS	Liver ex vivo signal	Lung ex vivo signal	Bowel ex vivo signal	Figure reference
PDOX-1	Week-based follow-up; exact postimplantation days unavailable	Clinical deterioration; euthanasia at study endpoint	Yes	Yes	Yes	No	—
PDOX-2	Weeks 2, 4, 6, 8, 9, and 10; exact postimplantation days unavailable	Clinical deterioration; euthanasia at study endpoint	Yes	Yes	Yes	Yes	Figures 3 and 5
PDOX-3	Week-based follow-up; exact postimplantation days unavailable	Clinical deterioration; euthanasia at study endpoint	Yes	Yes	Yes	No	—
PDOX-4	Week-based follow-up; exact postimplantation days unavailable	Clinical deterioration; euthanasia at study endpoint	Yes	Yes	Yes	No	—
PDOX-5	Week-based follow-up; exact postimplantation days unavailable	Clinical deterioration; euthanasia at study endpoint	Yes	Yes	Yes	No	—
PDOX-6	Week-based follow-up; exact postimplantation days unavailable	Clinical deterioration; euthanasia at study endpoint	Yes	Yes	Yes	No	—
PDOX-7	Week-based follow-up; exact postimplantation days unavailable	Clinical deterioration; euthanasia at study endpoint	Yes	Yes	Yes	No	—

Note: All seven animals were initially monitored at approximately two-week intervals, with imaging shortened to approximately weekly intervals during the final phase because of deterioration in clinical condition. All animals reached the experimental endpoint on the same day because of weight loss, reduced activity, and anorexia. Exact postimplantation days and the numerical terminal day could not be reliably recovered from the available historical records and were therefore not retrospectively inferred. Figure 3 shows longitudinal in vivo imaging of PDOX-2, whereas Figure 5 shows endpoint ex vivo organ imaging from PDOX-2. The two liver panels in Figure 5 represent acquisitions of the two sides of the same liver.

 https://doi.org/10.3390/ijms27209051
